# Heart-Type Fatty Acid Binding Protein Level as a Tool in Identification of Early Cardiac Effects of Diabetic Ketoacidosis

**DOI:** 10.4274/jcrpe.3961

**Published:** 2017-06-01

**Authors:** Fatma Hilal Yılmaz, Sevil Arı Yuca, Hüsamettin Vatansev, Emine Ayça Cimbek, Yaşar Şen, İsa Yılmaz, Fikret Akyürek, Derya Arslan, Derya Çimen, Alaaddin Yorulmaz

**Affiliations:** 1 Selçuk University Faculty of Medicine, Department of Child Health and Diseases, Konya, Turkey; 2 Selçuk University Faculty of Medicine, Department of Pediatric Endocrinology, Konya, Turkey; 3 Selçuk University Faculty of Medicine, Department of Biochemistry, Konya, Turkey; 4 Konya Training and Research Hospital, Clinic of Pediatric Cardiology, Konya, Turkey; 5 Selçuk University Faculty of Medicine, Department of Pediatric Cardiology, Konya, Turkey; 6 Beyhekim State Hospital, Clinic of Child Health and Diseases, Konya, Turkey

**Keywords:** Heart-type fatty acid binding protein, child, diabetic ketoacidosis

## Abstract

**Objective:**

This study aimed to measure the serum levels of heart-type fatty acid binding protein (H-FABP) in patients presenting with diabetic ketoacidosis (DKA) and diabetic ketosis (DK) and to determine its role in identifying early-period cardiac ischemia.

**Methods:**

This prospective study included 35 patients diagnosed with DKA, 20 patients diagnosed with DK, and 20 control subjects. H-FABP, creatine kinase-MB (CK-MB), and troponin I levels were investigated at presentation in patients with DKA and DK and in the control group. H-FABP values were measured again after acidosis correction in the DKA patients.

**Results:**

No statistically significant differences were found with respect to troponin I and CK-MB within the groups. The H-FABP values of DKA patients at presentation were found to be significantly higher than those of DK patients and the control group (p=0.015). The H-FABP value of the DKA group was also found to be significantly higher than the value at hour 36 after acidosis correction (p=0.0001).

**Conclusion:**

We would like to propose H-FABP as a potential marker for indicating myocardial ischemia.

## What is already known on this topic?

It is well known that diabetic ketoacidosis (DKA) has acute and severe effects on the myocardium.

## What this study adds?

In terms of patient management, heart-type fatty acid binding protein (H-FABP) is superior in showing cardiac involvement in the ischemia phase in DKA and this makes it more valuable than other biomarkers. In our study, the decrease in H-FABP levels noted after improvement of the ketoacidosis state has shown that cardiac ischemia in the early stages of DKA can be reversed with proper treatment without advancing to necrosis. Repetitive attacks of DKA may increase the risk of cardiac necrosis in the early period.

## INTRODUCTION

Diabetic ketoacidosis (DKA) is an acute complication of type 1 diabetes mellitus (T1DM) and a significant cause of morbidity and mortality. It is the most frequent reason for hospitalization among children with T1DM. Despite the developments in treatment and management, DKA emerges as the first presentation of newly diagnosed diabetes, as well. This rate may vary among countries and is in the range of 15% and 67% (1). Cerebral edema occurs in 0.5%-0.9% of all episodes of DKA, and the most frequent cause of mortality is brain edema (2,3). Acute respiratory distress, hypo- or hyperkalemia, acute renal failure, and disseminated intravascular coagulation are some of the other life-threatening complications (4).

Heart-type fatty acid-binding protein (H-FABP) is a novel biomarker shown to be released from the injured myocardium and is detected in blood within 1 hour after the onset of ischemia (5). Several studies have demonstrated that it is a sensitive early marker of myocardial injury (6,7). Recent clinical and experimental studies have suggested that H-FABP is superior to creatine kinase-MB (CK-MB) or cardiac troponins for early detection of ischemic myocardial necrosis (6,8). H-FABP is a low molecular weight (14.5 kDa) protein which contains 132 amino acid residues. Fatty acid-binding proteins bind the long-chained fatty acids reversibly and non-covalently, facilitating the intracellular cytoplasmic transport of the fatty acids and are highly expressed in tissues with active fatty acid metabolism such as the heart muscle. Their concentration in blood reaches the maximum value within 6-8 hours and generally decreases within 24-36 hours (9).

Reports on the cardiac effects of DKA in children and adolescents are limited (10,11). In this study, the serum level of H-FABP, a cardiac-specific marker, was measured in pediatric and adolescent patients presenting with ketoacidosis and ketosis. The role of this protein in indicating early-period cardiac ischemia was demonstrated.

## METHODS

The study was conducted between 2013 and 2015 at the Pediatric Endocrinology Clinic of the Selçuk University School of Medicine Hospital as a prospective randomized controlled study. The study included 55 patients aged 1-18 years, divided into 35 patients with DKA and 20 patients with diabetic ketosis (DK). The control group comprised 20 volunteer healthy children and adolescents who presented for routine follow-up and who did not have any infectious or chronic diseases. The patients that presented with signs of diabetes received a diagnosis of DKA based on the following findings: plasma glucose level >200 mg/dL, pH<7.35, serum bicarbonate level <15 mEq/L, and presence of ketones in the urine and serum. The patients who had a plasma glucose level of >200 mg/dL and ketonemia or ketonuria but had pH>7.30 in venous blood and blood bicarbonate level >15 mEq/L were diagnosed as DK.

The H-FABP, CK-MB, and troponin I levels of the DKA and DK patients and the control group were determined at the time of presentation. For DKA patients, the H-FABP level was also measured again 36 hours after the correction of acidosis. Blood gases, serum bicarbonate, urea, creatinine, alanine transaminase (ALT), and aspartate aminotransferase (AST) levels, as well as hemograms, were also determined in the patient and control groups. The blood ketones were checked in the patient group.

The electrocardiographs (ECGs) of the patient and control groups were obtained with a Nihon-Kohden device. The assessment was made by a pediatric cardiologist based on the criteria for ischemia of a normal T axis with ST depression, T-wave inversion, and ST elevation in the left precordial leads, and the criteria for infarction of Q-wave longer than 0.03 seconds, ST elevation, and T-wave inversion.

The study was approved by the Ethics Committee of Selçuk University. Informed consent and data release approval forms were obtained from all individuals above 12 years of age and from the parents of all individuals below 12 years of age.

The study excluded patients with type 2 diabetes mellitus or monogenic diabetes, cardiovascular, hepatic, and other chronic diseases, a history of smoking, or a history of drug, substance, or alcohol abuse.

Hycult Biotech (USA) commercial kits were used to analyze H-FABP in a Rayto-2100C Microplate Reader (India) device via the enzyme-linked immunosorbent assay method. CK-MB and troponin I values were analyzed using a Hitachi Cobas 601 (Japan) device through the method of electroluminescence in Roche commercial kits. The hemograms were analyzed in an Abbott CELL-DYN 3700 System (Germany) device. Blood gas was analyzed in a Techno Media Gastat 604 ox (Japan) device. Urea, creatine, and transaminases were analyzed in an Abbott Architect C16000 (Japan) device via the colorimetric method in Abbott commercial kits. Electrolytes were analyzed in an Abbott Architect C16000 (Japan) device via the colorimetric method, using an Ion Selective Electrode (ISE) system in Abbott commercial kits.

### Statistical Analysis

For statistical analysis, the SPSS-15 (SPSS Inc., Chicago, Illinois, USA) software pack was used. For the evaluation of nominal distribution, the Kolmogorov-Smirnov test was applied. For data that were compliant with normal distribution, parametric tests, an ANOVA variance analysis, and a Tukey’s honest test were performed. For data that were not compliant with normal distribution, non-parametric tests, Kruskal-Wallis variance analysis and Mann-Whitney U-test, were performed. The difference in H-FABP levels at patient’s admission and at hour 36 after correction of acidosis in the DKA group was assessed using the paired-samples t-test. The significance level was considered to be p<0.05.

## RESULTS

Our study included 55 T1DM children and adolescents. In the group presenting with DKA, 17 were female and 18 were male; their mean age was 120.3±60 months. The DK group consisted of 7 female and 13 male subjects; their mean age was 122.1±60 months. The control group included 13 female and 7 male subjects, and their mean age was 108.4±71 months. The groups had a similar distribution in terms of age, sex, height, and weight (Table 1).

In Table 2, the laboratory results of patients with DKA, patients with DK, and the control group are given. A statistically significant difference (p<0.0001) was found among the groups in terms of plasma glucose, venous pH, serum bicarbonate, and blood ketones. There was no significant difference between the group with DKA and the group with DK regarding serum creatinine values (p>0.878). On the other hand, there was a significant difference in serum creatinine values between the DKA/DK groups and the control group (p<0.0001). The H-FABP levels of patients at presentation was found to be significantly higher (p=0.015) in the group with DKA as compared to the other groups. White blood cell count was found to be significantly higher (p<0.004) in DKA and DK patients as compared to the control group. Phosphate and AST levels, not shown in the table, were significantly higher in the control group as compared to other groups (DK and DKA)(p=0.05 and p<0.0001, respectively).

No statistical significance (p=0.457) was found with respect to troponin I; the values were 0.06±0.08 (DKA), 0.07±0.04 (DK), and 0.04±0.04 (controls), respectively. No statistically significant difference (p=0.229) was detected among groups with respect to CK-MB values, which were 1.48±0.91 (DKA), 1.55±0.9 (DK), and 2.09±1.37 (controls), respectively. Table 3 shows the H-FABP levels for the patients at the time of presentation and at hour 36 after the correction of acidosis. The value at the time of presentation was found to be significantly higher (p<0.0001). H-FABP level at the time of presentation was statistically significantly higher in the group with DKA as compared to the DK and control groups (p=0.015). H-FABP levels were 1.17±0.79, 0.79±0.5, and 0.69±0.36, respectively in these three groups. The H-FABP value of the DKA group at presentation was found to be higher at a statistically significant level as compared to the value at hour 36 after the correction of acidosis, and these values were 1.17±0.79 and 0.55±0.28, respectively (p=0.0001). The correlation between the H-FABP level at presentation and the demographic and laboratory values of the patients was also checked. H-FABP was identified to be moderately positively correlated with glucose level (r=0.335, p=0.013), corrected serum sodium level (r=0.386, p=0.004), white blood cell count (r=0.374, p=0.005), urea (r=0.34, p=0.010), and blood creatinine (r=0.444, p=0.00) levels.

Abnormal findings such as ischemia, necrosis, arrhythmia, or tachycardia were not detected in the ECGs of neither the patient nor the control groups.

## DISCUSSION

It is well known that DKA has acute and severe effects on the myocardium. Hyperglycemia and acidosis lead to osmotic diuresis, dehydration, and loss of essential electrolytes (2). Thus, ischemia and myocardial cell damage occur with the disruption of tissue perfusion. Cardiac biomarkers are important in determining the risk of cardiac ischemia and necrosis, and in stopping their progression. The biomarkers which have been defined to show an increase in the blood especially during the course of cardiac ischemia and necrosis are rapidly released into the bloodstream after myocardial injury. The myocardial-specific troponin is used in correlation with CK and myoglobin because it is still used as the golden standard in this fiel d, and it increases 6 hours after the cardiac event. Although CK-MB levels increase significantly in 6-8 hours and myoglobin levels increase significantly 3 hours after myocardial infarction, they are not cardiac-specific markers and cannot distinguish myocardial damage from skeletal muscle injury (12). In studies on the myocardial-specific biomarkers, H-FABP is a new and reliable predictor to be included in the literature because its blood level increases within minutes after the initiation of the event and returns to baseline levels 36 hours later, and is at high levels only in the cytoplasm of the myocardial cells (13).

H-FABP is a cytosolic protein which is found in the cytoplasm of the cardiac myocytes and in the liver. It regulates the gene regulation of intracellular transport of fatty acid in ischemic process and also is considered to protect the cardiac myocytes against detergent-like effects (14). It is known to be excreted unchanged by the kidney (8). The small molecular weight protein, which is involved in intracellular transport of fatty acids, is used as a new marker determining recent myocardial damage. This molecule, which is released into the circulation at the first hour of the damage to the myocardial cell, achieves a peak within an average time of 4 hours (3-8 hours) and returns to normal levels within 12-36 hours (8,9).

The peak times of H-FABP, CK-MB, troponins I and T, which are used as specific markers of myocardial necrosis, are 3-12 hours, 4-12 hours, 12-18 hours and 12-48 hours, respectively (15). Therefore, H-FABP, troponin-I and CK-MB were analyzed together in our study. In a meta-analysis of 16 studies conducted by Azzazy et al (13), it was found that with a combination of H-FABP with other markers, its efficacy as a marker became more significant in the early phase of cardiac ischemia. The levels of H-FABP, CK-MB, and troponin-I were examined in adult patients admitted to the emergency department with carbon monoxide poisoning by Açıkalın et al (16) and they have concluded that H-FABP was a significant biomarker to show cardiac ischemia in the early period. Daly et al (17) emphasized that H-FABP was a valuable marker in the early diagnosis of acute myocardial infarction in 407 adult patients who were admitted to the emergency room with ischemic chest pain and had a negative troponin T result. Bank et al (18) agreed that H-FABP testing improves diagnostic accuracy when used in addition to clinical findings and ECG but stated that it had no additional diagnostic value when hs-cTnT measurements are also available. In the light of several studies performed, it is possible to say that H-FABP is a new and reliable marker in the detection of cardiac ischemia and necrosis.

In our study, H-FABP level was found to be higher at a statistically significant level at admission in patients with DKA compared to the other groups and this finding supports the opinion that acidosis causes cardiac ischemia. In the study performed by Atabek et al (10) to determine the cardiac effects of ketoacidosis in children, troponin I, CK-MB, and myoglobin levels were found to be significantly higher in the group with ketoacidosis. In our study, troponin I level was not higher at a statistically significant level in this group. Nevertheless, H-FABP level being higher at a statistically significant level in the group with DKA suggested that it was a more sensitive marker in ischemia and also that our patients were diagnosed in the early stage prior to the development of necrosis as a result of prolonged acidosis. We think that the possibility to determine patients at the ischemia stage with H-FABP is important in shaping the treatment and management of DKA.

The presence of high H-FABP levels at a stage where troponin I is not affected has demonstrated once again that it is a valuable and usable marker in determining ischemia which has not yet progressed to cause myocardial damage and necrosis. In our study, troponin I level was found to be low. 

In the literature, there are studies conducted on adults investigating the effects of DKA on the heart. In a study performed by Al-Mallah et al (19) in 2008, it was reported that troponin I level was high at admission in 26 of 96 patients with ketoacidosis and that patients with a high troponin I level followed for 2 years carried a high risk of cardiovascular disease and mortality. The authors emphasize that it is important to follow such patients for a long term for cardiovascular diseases. Long-term follow-up requirement is also one of the messages of our study which consisted of child and adolescent age groups with a longer life expectancy. Mollera et al (20) found that troponin I and CK-MB levels were high in two adult patients who were admitted with severe ketoacidosis in 2005 and who had no history of serious cardiovascular disease. Myocardial infarction findings were detected in the ECG of both patients. However, these authors have concluded that this situation developed with the release of enzyme-linked membrane instability caused by acidosis and the increased levels of free fatty acids, since the coronary arteriography of these patients was normal (20). George et al (21) made a comparison between patients with DKA and patients with hyperglycemia without ketoacidosis and reported that the mean systolic rate and pre-ejection period/left ventricular ejection times were significantly lower in the group with DKA, but that there was no difference between the two groups in terms of heart rate or arterial pressure.

Hyperglycemia causes abnormalities in calcium homeostasis and lipid metabolism with an increase in the level of free fatty acids and growth factors. Moreover, it is known to cause abnormal gene expression, improper signal transmission, and cardiomyocyte apoptosis due to oxidative stress by increasing the release of reactive oxygen molecules (22). In our study, although the patients with DK were hyperglycemic, their H-FABP level was found to be low. We suggest that while the effect of acidosis on the myocardium is mostly associated with ischemia in the acute phase, hyperglycemia has a cardiomyopathic effect in the chronic phase.

The lack of a statistically significant difference between the groups with and without DKA in terms of creatinine levels was explained by the dehydration present in both groups. The statistically significant difference at admission between the two groups in terms of H-FABP levels has suggested that the molecule is not affected by dehydration and also that ischemia is affected by acidosis rather than glucose elevation and dehydration. The statistically significant difference between the groups in terms of white blood cell counts was explained by the dehydration in the group with and without DKA. The low phosphorus level in the group with DKA was an expected finding. During DKA, the detection of a moderate positive correlation between the H-FABP level at admission and the plasma glucose level and also that between the H-FABP level and the corrected serum sodium levels, white blood cell, urine, blood creatinine, has been associated with prolonged duration of DKA and the severity of ischemia increased by acidosis. The presence of significantly high AST levels in the control group has ruled out the idea that H-FABP was elevated due to the possible hepatic ischemia in the group with ketoacidosis. On the other hand, the lack of need for intensive care and follow-up in our patients who were in a state of clear consciousness has ruled out the idea that the increase in H-FABP is due to cerebral ischemia. Thus, our results support the conclusion that H-FABP may be a biomarker of cardiac ischemia in DKA.

It has been emphasized that ketoacidosis and acidosis can cause a prolonged QT resulting in sudden cardiac death, and that cardiac monitoring is important in patients with DKA (23). In our study, even the ECG tracings of patients with DKA who had high H-FABP levels were normal. The lack of ischemia or infarct signs on ECG taken at the admission in the patient and control groups has suggested that in the early stages of ketoacidosis, cardiac ischemia may be asymptomatic. However, it is important to follow up the possible complications of cardiac ischemia with cardiac monitoring in patients with DKA in the acute phase. The lack of ischemia signs on ECG reveals that checking biochemical markers simultaneously is needed as well.

In conclusion, our findings indicate that H-FABP, which detects cardiac involvement in the ischemia phase in DKA, is an important tool in patient management. In our study, a decrease in H-FABP levels following the improvement of ketoacidosis has shown us that cardiac ischemia in the early stages of DKA can be reversed with proper treatment without progressing to necrosis. Repetitive attacks of DKA may increase the risk of cardiac necrosis in the early period.

## Figures and Tables

**Table 1:**
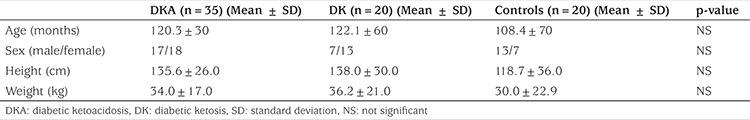
The demographic characteristics of patients with diabetic ketoacidosis, diabetic ketosis, and the control group

**Table 2:**
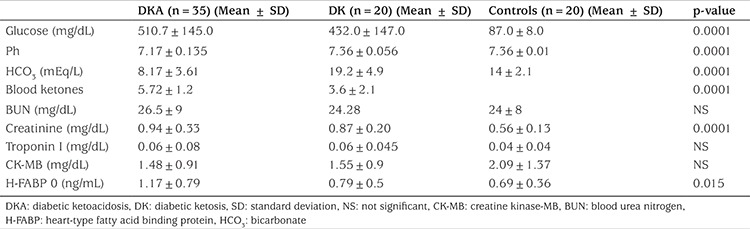
The laboratory results of patients with diabetic ketoacidosis, patients with diabetic ketosis, and the control group

**Table 3:**
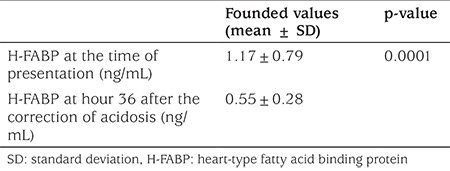
The comparison of heart-type fatty acid binding protein levels at the time of presentation of patients and at hour 36 after the correction of acidosis
